# Sub-cutaneous Syringe

**Published:** 1869-03

**Authors:** 


SUB-CUTANEOUS SYRINGE.—We have received from W. F. Ford, surgical instrument maker, 85 Fulton street, New York, a sub-cutaneous syringe, worthy of attention by the profession. It is according to the experience of physicians generally to find when they most want these instruments that they are out of working order. The rubber ones break so easily and are so constantly requiring repair that their use has been almost abandoned. The cheap glass instruments are not worthy even a mention; most of them cannot be used once. Sub-cutaneous medication is established as a necessity, and the manufacture of an instrument which will answer any reasonable expectation is a great advantage in its adoption. The one we have received from Mr. Ford appears to us to combine all the desirable qualities, strength, finish of workmanship, and absolute perfection in easily determining the number of drops administered. We regret our inability to state the price, but no matter what it is, a good instrument of the sort is cheap, at any price, compared with a poor one.

